# A Deep-Learning Approach toward Rational Molecular Docking Protocol Selection

**DOI:** 10.3390/molecules25112487

**Published:** 2020-05-27

**Authors:** José Jiménez-Luna, Alberto Cuzzolin, Giovanni Bolcato, Mattia Sturlese, Stefano Moro

**Affiliations:** 1 Department of Chemistry and Applied Biosciences, RETHINK, ETH Zuerich, Vladimir-Prelog-Weg 4, 8093 Zuerich, Switzerland; 2 Institute for Pure & Applied Mathematics, University California Los Angeles, 460 Portola Plaza, Los Angeles, CA 90095-7121, USA; 3 Molecular Modeling Section, Department of Pharmaceutical and Pharmacological Sciences, University of Padova, 35131 Padova, Italy; cuzzolin.alberto@gmail.com (A.C.); giovanni.bolcato.1@phd.unipd.it (G.B.); mattia.sturlese@unipd.it (M.S.)

**Keywords:** deep learning, structural biology, chemoinformatics, molecular docking

## Abstract

While a plethora of different protein–ligand docking protocols have been developed over the past twenty years, their performances greatly depend on the provided input protein–ligand pair. In this study, we developed a machine-learning model that uses a combination of convolutional and fully connected neural networks for the task of predicting the performance of several popular docking protocols given a protein structure and a small compound. We also rigorously evaluated the performance of our model using a widely available database of protein–ligand complexes and different types of data splits. We further open-source all code related to this study so that potential users can make informed selections on which protocol is best suited for their particular protein–ligand pair.

## 1. Introduction

Molecular docking is nowadays a common approach in a computational drug discovery pipeline [1,2]: knowing a good approximation to the crystal pose of a ligand can provide medicinal chemists with new ideas for lead optimization that could potentially accelerate structure-based drug design. A docking protocol can be described as the combination of a search algorithm that samples the conformational space of a ligand within a binding site and a scoring function, which quantitatively evaluates the accuracy of such poses.

While in many cases the conformational search operated by docking protocols is effective in producing the correct pose for a ligand (i.e., the crystallographic pose is generally reproduced within reasonable accuracy), scoring functions often fail in ranking them (i.e., the crystallographic pose often is usually not the one with the best score) [3]. Given that the choice of the scoring function considerably affects results, and, to rationalize protocol choice, the comparison of the performance of different protocols is commonly performed in the early stages of docking studies. In particular, the DockBench platform [4] was recently developed with the aim to facilitate protocol selection. The aforementioned platform presents a benchmark of different docking protocols in a self-docking routine, whose goal is to reproduce the pose of a ligand with a known co-crystal: the ability of each protocol in producing the crystallographic pose being measured in terms of their Root Mean Square Deviation (RMSD). In particular, the average and the lowest RMSD (${\mathrm{RMSD}}_{\mathrm{ave}}$ and ${\mathrm{RMSD}}_{\min}$) of the generated poses are reported, as well as the number of poses with a lower RMSD than the X-ray resolution of the corresponding crystal (*n*RMSD) [5]. The success of introducing a benchmarking procedure in molecular docking campaigns has been reported in several blind challenges [6,7]. This approach has been shown to be particularly useful when multiple protein–ligand complexes are available for the same target, making protein conformation choice a further variable to be considered.

An ideal docking scoring function would produce the lowest ${\mathrm{RMSD}}_{\mathrm{ave}}$ and ${\mathrm{RMSD}}_{\min}$ metrics, leading to a better reproduction of the crystallographic pose. Motivated by this and the previously mentioned challenges, in the work presented here, we try to address the following two questions:

1. Given a particular docking protocol, would it be possible to know a priori which protein–ligand pairs will result in the best docking pose?
2. Is there a preferable way of choosing the best docking protocol for an arbitrary ligand rather than selecting the one that reproduces the best self-docking pose for a particular proteins structure?

Applications of Deep Learning (DL) in drug discovery have become ubiquitous in the last few years, as these methods have shown promise in relevant problems such as property prediction [8,9,10,11,12,13], compound retrosynthesis [14], de-novo drug design [15,16], and reaction prediction [17], among many others.

In the context of molecular docking, DL approaches have been investigated to replace classical scoring functions, showing moderate success [18,19], but still far behind the accuracy provided by standard docking procedures. Partially due to this fact, in this study, we explored the potential of DL approaches to both select the best possible docking protocol given a protein–ligand pair and to provide insight into which protein–ligand pairs will result in a better pose given a docking protocol. We performed an exhaustive evaluation of the proposed methodology using the diverse and well-known PDBbind protein–ligand database [20] and different data splits to conclude that the approach is able to help users make informed docking modeling choices. We furthermore open-source all our production and evaluation code so that the community can either use our models or reproduce the results presented in this work easily.

## 2. Results and Discussion

We prepared the protein–ligand refined set of the PDBbind database [21] (v.2017) according to the workflow previously described in the DockBench suite (see Section 3.1 and Section 3.2). With these data, we used the aforementioned software to generate docking results for 14 different well-known commercial and open-source protocols (see Section 3.3). A combination of 3D-convolutional and fully connected neural networks (see Section 3.5) was used as our main model alongside a voxelized representation of the protein pocket and a mixture of extended connectivity fingerprints [22] and two-dimensional descriptors for the ligand (see Section 3.4). The proposed model was trained to predict three quantities of interest (${\mathrm{RMSD}}_{\mathrm{ave}},{\mathrm{RMSD}}_{\min}$, and nRMSD) with the goal of determining which protein–ligand pairs work better under specific docking protocols (i.e., our first research question). We furthermore used four different evaluation data splits (see Section 3.6) to understand under which circumstances the models here presented perform optimally. For each docking protocol (see Section 3.3), we present results on the evaluation of the predicted ${\mathrm{RMSD}}_{\mathrm{ave}},\;{\mathrm{RMSD}}_{\min}$, and nRMSD against the molecular docking results, using the root mean squared error (RMSE) and Pearson’s correlation coefficient *R* metrics (Table 1 and Tables S1 and S2).

We first focus on the comparison between the random and ligand scaffold splits, arguably the most commonly used evaluation procedures in other chemoinformatics ML-based studies. Results for the random split show moderately good results, with some docking protocols showing average correlations over 0.6 (autodock-ga, autodock-lga, gold-asp, gold-chemscore, and gold-plp), suggesting that for those it is easier to predict which ligands will result in a better docking pose. On the other hand, results are significantly worse for the ligand-scaffold-based split for most protocols, which suggests that it is significantly harder for the model to distinguish which compounds outside the training set chemical manifold will result in a better docking result. This conclusion is in line with other works, where random-split-based results were significantly better than those provided by more sophisticated alternatives, such as the ligand-scaffold-based one [13,23,24].

Given that docking is inherently a structure-based problem, we also decided to explore model performance under different protein-dependent splits. The first protein-based split separates samples into different non-overlapping PFAM clusters (here named protein classes), showing a similar performance to the random split, albeit slightly inferior, suggesting that, while protein information plays a role, wider sampling of ligand chemistry space during training may have a more relevant impact. In the last type of split we evaluated, we sampled for training a percentage of complexes belonging to each protein family (protein classes balanced): our reasoning was that having a more homogeneous sampling of protein space would show a significant performance improvement.

**Figure 1:**
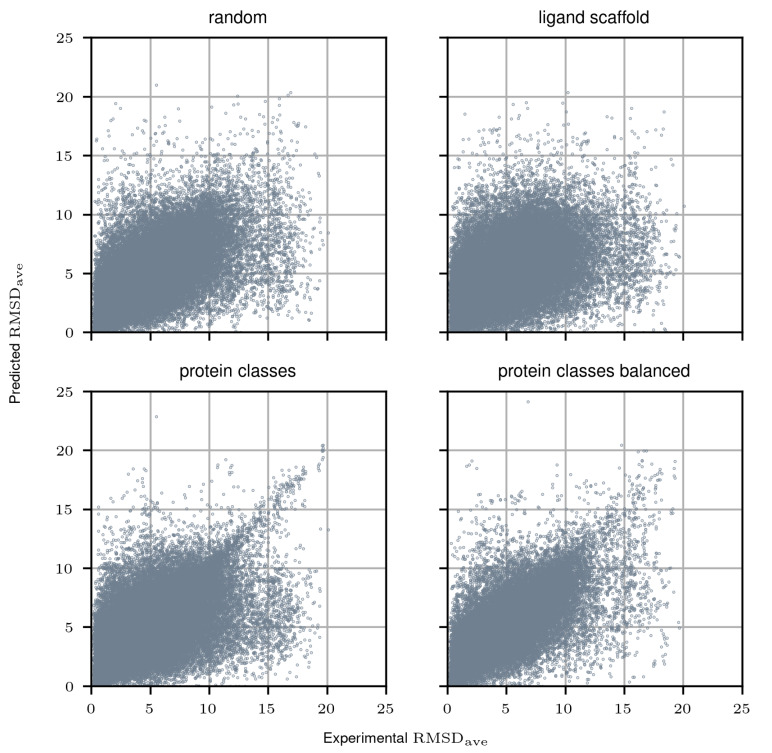
Ligand-centric ${\mathrm{RMSD}}_{\mathrm{ave}}$ evaluation merging all protocols and for all different types of proposed splits.

**Figure 2:**
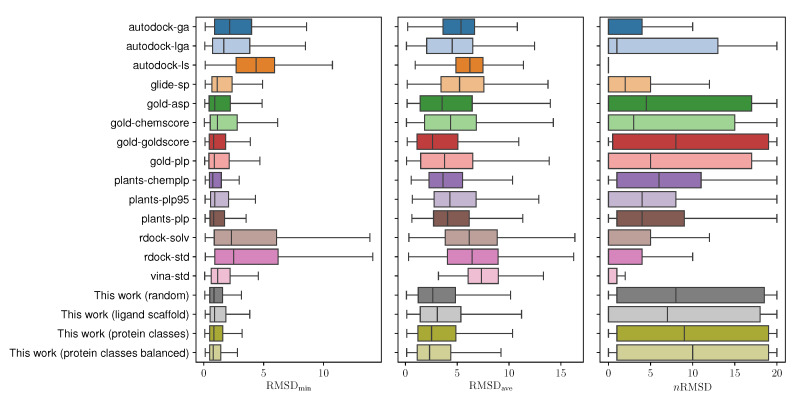
Distribution of ${\mathrm{RMSD}}_{\min}$, ${\mathrm{RMSD}}_{\mathrm{ave}}$, and nRMSD values in a self-docking scenario using the PDBbind v.2017 database of cocrystals, for all the protocols described in Table 3, and the approach proposed in this work under different evaluation scenarios.

Further evaluation was considered to tackle our second research question, the capability of the proposed model to choose the optimal docking protocol given a particular protein–ligand pair. Results can be consulted in Table 2 and Tables S3 and S4 as well as in Figure 1, where we draw similar conclusions as in the protocol-centric evaluation, with the proposed model performing worse in the ligand scaffold split scenario than in the others. Furthermore, in Figure 2, we consider the distribution of the experimental ${\mathrm{RMSD}}_{\min}$, ${\mathrm{RMSD}}_{\mathrm{ave}}$, and nRMSD values had we followed the recommendations of the proposed model, with the intent of investigating whether in fact it produces protocol selections that may improve docking errors. For both ${\mathrm{RMSD}}_{\min}$ and ${\mathrm{RMSD}}_{\mathrm{ave}}$ values, the protocol with the minimum predicted value was selected, while for nRMSD the maximum was chosen—and then their corresponding experimental values were analyzed. With the exception of the ligand scaffold scenario, the decisions undertaken by the proposed model produce the lowest mean ${\mathrm{RMSD}}_{\min}$ and ${\mathrm{RMSD}}_{\mathrm{ave}}$, and the highest nRMSD values compared to the rest of the protocols. Additional significance analyses were performed with a unilateral two-sample Mann–Whitney test. Using a significance level of α=0.01, we can conclude that the procedure here proposed results in significantly lower ${\mathrm{RMSD}}_{\mathrm{ave}}$ values than the rest of the protocols in all the evaluation scenarios, with the notable exception of gold-goldscore, where no statistical conclusion could be drawn in any direction either in the random, ligand scaffold, and protein clases splits. Interestingly, in the balanced protein split scenario, our approach manages to significantly outperform the aforementioned protocol.

**Figure 3:**
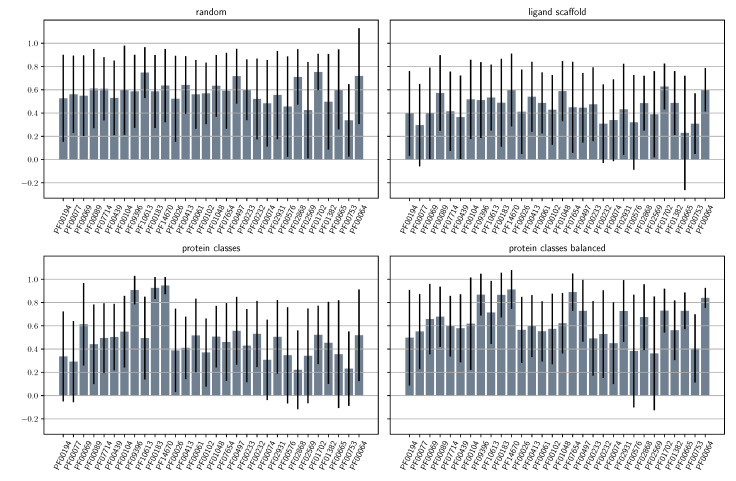
Average Pearson’s *R* correlation coefficient for the ${\mathrm{RMSD}}_{\mathrm{ave}}$ metric for all types of splits disaggregated into the 30 most populated PFAM families in the PDBbind refined dataset.

Overall results suggest that the proposed model provides better suggestions if both ligand chemistry and protein families are not significantly far from the training set manifold. We also investigated disaggregated performance for the 30 most populated PFAM families in our dataset (Figure 3 and Figure S1), to find similar conclusions to the previous evaluations. The results show that the model performs similarly well for the most populated families, and particularly for those splits that more uniformly sample protein space (i.e., the random and protein classes balanced), again highlighting the importance of structure-based models.

## 3. Materials and Methods

In this section, we first describe the preprocessing procedure for the complexes considered in this study as well as the docking simulation setup. We then describe the two different types of features used and the proposed neural-network architecture. Finally, we discuss technical training details as well as the evaluation procedure undertaken.

### 3.1. Datasets

The complexes considered for this study were retrieved from the 2017 version of the PDBbind database [21] In particular, we focused on its refined set, that we recently used for a large docking benchmarking campaign [25]. It consists of 4463 protein–ligand complexes, although 294 protein–peptide complexes were excluded as they were not considered in the original DockBench study, resulting in a final dataset of 4169 complexes. Docking settings were selected so as to match as close as possible the default parameters provided by the developers of each protocol for the handling of small organic molecules.

### 3.2. Complex Preparation

The proteins in the complexes were prepared according to a protocol previously reported [25]. Structures were processed using an internal workflow written in Scientific Vector Language (SVL), based on the protein preparation tool included in the MOE molecular suite [26]. First, crystal structural issues such as missing atoms and partially solved residues were fixed, hydrogen atoms were added and protonation states for all titrable residues were computed. Finally, solvent molecules and impurities (e.g., co-solvents) were removed. An additional preparation step for the ligands was taken, in which the most favorable ionic state was calculated and partial charges of atoms were assigned. Towards this end, we take advantage of two tools provided by the OpenEye toolkit: fixpKa and molcharge [27]. Finally, ligand geometries were minimized before docking using Open Babel’s [28] routing and the MMFF94 force field [29].

### 3.3. Data Generation

The docking simulation and consequent data generation were performed via the DockBench software (version 1.06), which automates docking simulations and evaluates protocol performances in reproducing ligand conformations in the crystal structure. We included 14 docking protocols from six different software alternatives: AutoDock 4.2.5.1 [30], Vina 1.1.2 [31], PLANTS 1.2 [32], rDOCK [33], Glide 6.5 [34], and Gold 5.4.1 [35]. For each of the included protocols, we defined the binding site as a sphere of a 15Å radius centered at the center of mass of the co-crystalized ligand, and we generated 20 poses with an RMSD separation of at least 1Å. In the case of both Autodock and Vina, since they do not support spheric site definition, the cube side is scaled to $\sqrt{\frac{4\pi }{3}r}$ to maintain comparable volumes with the protocols adopting parallelepiped-shaped cavity definitions, where *r* is the sphere radius. In addition, in the case of Vina, to guarantee that at least 20 poses were returned, we modified the “maximum energy difference” argument. Description of the protocols, as well as their search algorithms and scoring functions can be found in Table 3.

We studied three different and complementary evaluation values for prediction as described in the DockBench suite: the minimum RMSD (${\mathrm{RMSD}}_{\min}$), the average RMSD (${\mathrm{RMSD}}_{\mathrm{ave}}$) and the number of poses with an RMSD lower than the resolution of their corresponding crystal structures (nRMSD). Box plots detailing the distribution of these values are available in Figure 2, where we can clearly highlight that some protocols (e.g., gold-asp, gold-goldscore, gold-plp, or glide-sp) display consistent accuracy in many benchmark scenarios, while others (e.g., rdock-solv and autodock-lga) display a higher error variability depending on the input.

### 3.4. Descriptor Calculation

We take a structure-based approach to represent proteins, deciding to use 3D-voxel descriptors [36,37] that capture the influence of each atom to each voxel of the grid via a pair correlation function n(r) that depends on their euclidean distance *r* and the Van der Waals radius $r_{\mathrm{vdw}}$ of the first:

(1) $$n\left(r\right)=1-\exp\left(-{\left(\frac{r_{\mathrm{vdw}}}{r}\right)}^{12}\right).$$

We used the voxelization routines available in the HTMD python framework for molecular modeling [38], which computes eight different pharmacophore-like properties: hydrophobic, aromatic, hydrogen-bond acceptor and donor, positive and negative ionizable, and metallic and total excluded volume. A 24 Å${}^{3}$ array was computed and centered on the center of mass of the co-crystalized ligand, with a resolution of 1 Å. For the ligands, we used Extended Connectivity Fingerprints (ECFP4) [22] with a size of 1024 bits and a radius of 2 bonds as well as a set of 183 physical-chemical descriptors available in the RDKit software [39].

### 3.5. Neural Network Architecture

A Neural Network (NN) architecture usually takes an array-based input and performs several transformations to obtain another array-based output [40]. Depending on the nature of the input array, some architectures are more appropriate than others. For instance, when the input represents a spatial arrangement (e.g., an image or the 3D-voxel representation described here), a convolutional neural network (CNN) is a typical choice, whereas a fully forward neural network (FNN) is more suitable for a one-dimensional vector, such as a chemical fingerprint [41]. In this study, we designed a specific neural network that takes advantage of both CNN and FNN architectures so as to handle both input types appropriately.

**Figure 4:**
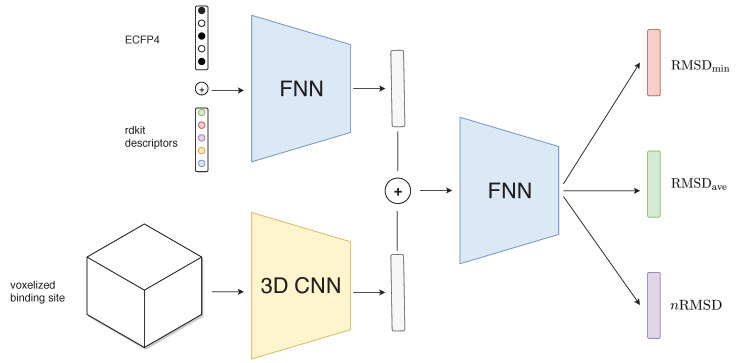
Schema of the proposed architecture in this work. A fully connected neural network handles ECFP4 fingerprints and descriptors computed from RDKit while a 3D-convolutional neural network processes a voxelized representation of the protein binding site. Latent space from both inputs is then concatenated and fed into further fully connected layers that predict the three outputs of interest per docking protocol.

We designed a two-legged neural network that takes protein voxels and ligand fingerprints as inputs separately (Figure 4). Protein voxels pass through five convolutional layers with a rectified linear unit activation function and then they are flattened into a one-dimensional vector. In parallel, ligand descriptors are fed to three consecutive linear layers again with the ReLU activation function. Then, the outputs of both legs are concatenated into a single vector of size 1024. A batch normalization layer [42] is then applied to this hidden protein–ligand representation and three different output linear layers with ReLU activation function are computed, corresponding to each of the three metrics used by DockBench: ${\mathrm{RMSD}}_{\min}$, ${\mathrm{RMSD}}_{\mathrm{ave}}$ and *n*RMSD. For the first two RMSD-based outputs, we used a standard mean-squared-error loss, while, for *n*RMSD, we use a Poisson negative log-likelihood loss function, defined by:

(2) $$\ell \left(y,\hat{y}\right)=\hat{y}-y\log\left(\hat{y}\right)+\log\left(y!\right),$$

where *y* and $\hat{y}$ are true and predicted values, respectively. We consider the unweighted sum of these three objectives for loss minimization.

### 3.6. Training and Validation

We used a *k*-fold cross-validation scheme (k=5) to estimate model performance under different split scenarios: for each split, a model is trained on k−1 non-overlapping subsets and evaluated on the remaining one. Furthermore, we decided to investigate the dependency of the performance with respect to the composition of the chosen subsets. For this reason, we considered four different sampling procedures, each representing a particular application scenario: (i) a completely random split; (ii) a ligand-scaffold-based split where compounds are grouped according to a *k*-means clustering of the ligands’ ECFP4 fingerprints [43]; (iii) a protein-based split based on non-overlapping PFAM families [44]; and (iv) a balanced protein-class-based split, where we randomly sample 20% of the validation complexes from each PFAM family. In each of the splits, we trained the model for 200 epochs using the Adam optimizer [45] ($\beta_{1}=0.99,\beta_{2}=0.999$) with a starting learning rate of $10^{-3}$ coupled with an exponential learning rate scheduler (γ=0.95) and a batch size of 32 samples. Data augmentation was performed during training by applying random rotations to the protein pocket coordinates using the geometric center of the ligand as point of reference.

### 3.7. Implementation and Code Availability

The final production model as well as code to train it and replicate all results and analyses in this paper are openly available on a GitHub repository (github.com/cuzzo87/CNNDockBench) under an AGPLv3 license. Users can easily use production model scripts to run predictions for their protein–ligand pairs. Our model was implemented in Python using PyTorch (version 1.0) [46] as our main tensor manipulation and automatic differentiation library. While GPU support is not needed for the replication of our work, as well as its production usage, it is strongly recommended, as it can substantially accelerate computations.

## 4. Conclusions

In this study, we developed a deep-learning-based pipeline for the informed selection of a particular molecular docking protocol, given a protein–ligand pair, and the elucidation of which protein–ligand pairs result in a better pose with a predefined docking algorithm. In conclusion, we believe that we successfully managed to answer both of those research questions. First, we show that it is possible to predict which protein–ligand pairs produce the best poses given a particular docking protocol, although results greatly vary depending on the latter. Interestingly, some protocols (autodock-ga, autodock-lga, gold-asp, and gold-plp) show easier predictability across different data splits than others (plants-plp95, plants-plp, rdock-solv, and rdock-std). We also show that it is certainly possible to predict which docking protocols are better suited for a given protein–ligand pair using the proposed model, although predictive performance greatly depends on the type of the evaluation split taken. Specifically, performance on the random and balanced protein classes splits is undoubtedly superior to that on the ligand scaffold split in most of our evaluations. In addition, we measured the distribution of several relevant docking-related metrics according to the suggestions of the proposed methodology, to find that these are consistently better than other existing individual protocols under most circumstances.

In general, the results presented in this work highlight the usefulness of the presented methodology, but also show that its performance greatly varies depending on the type of evaluation split taken, suggesting that its prospective applicability may differ depending on how close both protein and ligand queries are to the training set manifold. Along those lines, we believe that future interested users in the proposed approach should take these points into consideration before evaluation or re-training of the neural network on their own data. Additionally, while we thoroughly benchmarked our model, all the evaluations presented here are retrospective per se. Future blind structure-based evaluations, such as the ones proposed by the D3R Grand Challenges [47,48,49], would provide excellent opportunities to evaluate approaches similar to the one proposed here prospectively.

Methodology-wise, there are several interesting directions for future research regarding neural network architectural design. In particular, it is a well-known issue that 3D-convolutional neural networks are not rotationally equivariant [50] (i.e., the output of the network varies if the coordinates of the protein are rotated), a desirable characteristic when modeling atomistic systems. While this issue is mitigated in the current work through data augmentation, recent approaches such as SE(3) equivariant neural networks [51] bear promise towards solving this issue. On the ligand side, graph convolutions [52] are a family of approaches that are displaying good results in a variety of tasks relevant to drug discovery, such as property prediction [11,12,53] or compound generation [54]. How these approaches would perform in the task proposed here remains a topic for further exploration.

Finally, while we firmly believe that future-generation docking protocols will more tightly incorporate machine-learning elements into their pipelines [18,19] (e.g., by the design of more efficient search algorithms or scoring functions [55,56]), we think that the approach proposed in this paper represents a novel research direction that will drive structure-based drug design researchers towards more rational existing docking protocol choices. Hence, with the intent of improving research reproducibility and lowering accessibility barriers, we have open-sourced all evaluation and deployment code as well as trained models related to this work.

## Figures and Tables

**Table 1:** Predictive performance for ${\mathrm{RMSD}}_{\mathrm{ave}}\;\left(\pm 1\;\mathrm{std}.\right)$ per docking protocol, for each of the four splits considered.

Protocol	RMSE	Pearson’s *R*	RMSE	Pearson’s *R*	RMSE	Pearson’s *R*	RMSE	Pearson’s *R*
	Random		Ligand Scaffold		Protein Classes		Protein Classes Balanced	
autodock-ga	1.60 (±0.08)	0.74 (±0.03)	1.34 (±0.26)	0.38 (±0.21)	1.76 (±0.09)	0.60 (±0.05)	1.48 (±0.04)	0.73 (±0.02)
autodock-lga	2.01 (±0.08)	0.65 (±0.03)	1.82 (±0.41)	0.30 (±0.20)	2.20 (±0.13)	0.57 (±0.05)	1.89 (±0.03)	0.70 (±0.02)
autodock-ls	2.04 (±0.09)	0.50 (±0.04)	1.79 (±0.18)	0.50 (±0.14)	2.02 (±0.05)	0.41 (±0.04)	1.93 (±0.03)	0.46 (±0.02)
glide-sp	2.79 (±0.18)	0.52 (±0.05)	3.34 (±0.55)	0.14 (±0.14)	2.84 (±0.38)	0.44(±0.07)	2.34 (±0.12)	0.64 (±0.03)
gold-asp	2.43 (±0.10)	0.68 (±0.02)	2.50 (±0.58)	0.50 (±0.21)	2.52 (±0.21)	0.64 (±0.14)	2.08 (±0.08)	0.78 (±0.01)
gold-chemscore	2.59 (±0.14)	0.62 (±0.03)	2.74 (±0.39)	0.37(±0.19)	2.62 (±0.12)	0.61 (±0.03)	2.25 (±0.13)	0.73 (±0.02)
gold-goldscore	2.47 (±0.10)	0.52 (±0.03)	2.44 (±0.72)	0.53 (±0.29)	2.49 (±0.19)	0.51 (±0.06)	2.12 (±0.14)	0.66 (±0.03)
gold-plp	2.49 (±0.15)	0.66 (±0.03)	2.53 (±0.52)	0.32 (±0.22)	2.57 (±0.27)	0.62 (±0.06)	2.14 (±0.05)	0.76 (±0.01)
plants-chemplp	2.55 (±0.17)	0.44 (±0.02)	2.68 (±0.99)	−0.02 (±0.06)	2.55 (±0.24)	0.56 (±0.23)	2.23 (±0.13)	0.58 (±0.02)
plants-plp95	3.04 (±0.09)	0.42 (±0.02)	3.16 (±0.89)	−0.12 (±0.07)	3.08 (±0.23)	0.40 (±0.03)	2.58 (±0.22)	0.57 (±0.04)
plants-plp	2.75 (±0.17)	0.43 (±0.02)	2.76 (±0.58)	0.09 (±0.37)	2.79 (±0.27)	0.41 (±0.28)	2.44 (±0.10)	0.54 (±0.02)
rdock-solv	3.95 (±0.23)	0.35 (±0.26)	3.58 (±0.34)	0.09 (±0.08)	3.73 (±0.48)	0.42 (±0.09)	3.33 (±0.22)	0.54 (±0.18)
rdock-std	3.92 (±0.05)	0.35 (±0.25)	3.62 (±0.43)	0.08 (±0.46)	3.71 (±0.41)	0.42 (±0.09)	3.23 (±0.19)	0.56 (±0.03)
vina-std	2.23 (±0.03)	0.40 (±0.03)	2.30 (±0.15)	0.19 (±0.38)	2.35 (±0.16)	0.33 (±0.06)	1.97 (±0.12)	0.69 (±0.05)
**Average**	2.63 (±0.63)	0.52 (±0.11)	2.62 (±0.71)	0.24 (±0.16)	2.66 (±0.57)	0.50 (±0.12)	2.29 (±0.48)	0.64 (±0.10)

**Table 2:** Ligand-centric evaluation (${\mathrm{RMSD}}_{\mathrm{ave}},\pm 1\;\mathrm{std}.$) for the four different proposed split types in this study.

Split Type	Pearson’s *R*	RMSE
random	0.54(±0.01)	2.47(±0.05)
ligand scaffold	0.47(±0.07)	2.58(±0.64)
protein classes	0.56(±0.04)	2.33(±0.21)
protein classes balanced	0.65(±0.01)	1.98(±0.07)

**Table 3:** Docking protocols, search algorithms, and scoring functions considered in this study.

Score	Search Algorithm	Scoring Function	Protocol Abbrv.
Autodock 4.2	Local search	Autodock SF	autodock-ls
	Lamarckian GA		autodock-lga
	GA		autodock-ga
Glide 6.5	Glide algorithm	Standard precision	glide-sp
GOLD 5.4.1	GA	ASP	gold-asp
		Chemscore	gold-chemscore
		Goldscore	gold-goldscore
		PLP	gold-plp
PLANTS 1.2	ACO algorithm	ChemPLP	plants-chemplp
		PLP	plants-plp
		PLP95	plants-plp95
rDock 2013.1	GA + MC + Simplex minimization	rDock master SF	rdock-std
		rDock master SF + desolvation	rdock-solv
Vina 1.1.2	MC + BFGS local search	Vina SF	vina-std

GA (Genetic Algorithm), MC (Monte Carlo), BFGS (Broyden–Fletcher–Goldfarb–Shanno), ASP (Astex Statistical Potential), PLP (Pairwise Linear Potential), ACO (Ant Colony Optimization).

## Supplementary Materials

The following are available online, Figure S1: Average RMSE for the ${\mathrm{RMSD}}_{\mathrm{ave}}$ metric for all types of splits disaggregated into the 30 most populated PFAM families in the PDBbind refined dataset. Table S1: Predictive performance for ${\mathrm{RMSD}}_{\min}\;\left(\pm 1\;\mathrm{std}.\right)$ per docking protocol, for each of the four splits considered. Table S2: Predictive performance for n${\mathrm{RMSD}}_{,}\left(\pm 1\;\mathrm{std}.\right)$ per docking protocol, for each of the four splits considered. Table S3: Ligand-centric evaluation for the ${\mathrm{RMSD}}_{\min}\left(\pm 1\;\mathrm{std}.\right)$ metric and the four different proposed split types in this study. Table S4: Ligand-centric evaluation for the nRMSD(±1std.) metric and the four different proposed split types in this study.

## Abbreviations

The following abbreviations are used in this manuscript:

RMSD	Root mean squared distance
DL	Deep learning
NN	Neural network
CNN	Convolutional neural network
FNN	Fully-connected neural network
