# Supplementary material for: A Deep-Learning Approach toward Rational Molecular Docking Protocol Selection
Source: Molecules. 2020 May 27;25(11):2487. doi: 10.3390/molecules25112487 (PMC7321124; doi:10.3390/molecules25112487)
Supplement: Supplementary file 1 [file molecules-25-02487-s001.pdf]

A deep-learning approach toward rational molecular docking protocol selection. Supplementary information

José Jiménez-Luna,[*,†,‡,§] Alberto Cuzzolin,[¶,§] Giovanni Bolcato,[¶] Mattia Sturlese,[¶] and Stefano Moro[*,¶]

†*Department of Chemistry and Applied Biosciences, RETHINK, ETH Zürich, Switzerland.*

‡*Institute for Pure & Applied Mathematics, University California Los Angeles, 460 Portola Plaza, 90095-7121 Los Angeles, CA*

¶*Molecular Modeling Section, Department of Pharmaceutical and Pharmacological Sciences, University of Padova, 35131 Padova, Italy*

§*Equal contribution*

E-mail: jose.jimenez@rethink.ethz.ch; stefano.moro@unipd.it

List of Tables

1 Predictive performance for $\mathrm{RMSD_{min}}$ (± 1 std.) per docking protocol, for each of the four splits considered. 3

2 Predictive performance for nRMSD, (± 1 std.) per docking protocol, for each of the four splits considered. 3

3 Ligand-centric evaluation for the $\mathrm{RMSD_{min}}$ (± 1 std.) metric and the four different proposed split types in this study. 3

4 Ligand-centric evaluation for the nRMSD(± 1 std.) metric and the four different proposed split types in this study. 4

List of Figures

1 Average RMSE for the RMSD_{ave} metric for all types of splits disaggregated into the 30 most populated PFAM families in the PDBbind refined dataset. . 4

Table 1: Predictive performance for $\mathrm{RMSD_{min}}$ (± 1 std.) per docking protocol, for each of the four splits considered.

Protocol	RMSE	Pearson's R	RMSE	Pearson's R	RMSE	Pearson's R	RMSE	Pearson's R
	random		ligand scaffold		protein classes		protein classes balanced	
autodock-ga	1.74 (±0.06)	0.51 (±0.03)	1.85 (±0.24)	0.35 (±0.03)	1.81 (±0.18)	0.44 (±0.06)	1.64 (±0.04)	0.59 (±0.03)
autodock-lga	2.05 (±0.06)	0.45 (±0.04)	2.05 (±0.43)	0.25 (±0.14)	2.17 (±0.13)	0.35 (±0.07)	1.92 (±0.05)	0.54 (±0.04)
autodock-ls	2.37 (±0.07)	0.27 (±0.03)	2.22 (±0.16)	0.20 (±0.19)	2.24 (±0.13)	0.37 (±0.05)	2.29 (±0.03)	0.37 (±0.03)
glide-sp	2.32 (±0.08)	0.42 (±0.01)	2.34 (±0.69)	0.23 (±0.13)	2.34 (±0.17)	0.37 (±0.06)	2.06 (±0.18)	0.52 (±0.06)
gold-asp	1.97 (±0.15)	0.45 (±0.05)	1.82 (±0.94)	0.21 (±0.12)	1.99 (±0.14)	0.45 (±0.06)	1.81 (±0.10)	0.59 (±0.04)
gold-chemscore	2.35 (±0.12)	0.40 (±0.05)	2.18 (±0.89)	0.20 (±0.14)	2.27 (±0.14)	0.46 (±0.08)	2.12 (±0.12)	0.56 (±0.04)
gold-goldscore	1.98 (±0.15)	0.32 (±0.04)	1.70 (±0.70)	-0.04 (±0.48)	1.94 (±0.21)	0.35 (±0.09)	1.74 (±0.11)	0.59 (±0.21)
gold-plp	2.03 (±0.18)	0.47 (±0.06)	1.89 (±0.96)	0.22 (±0.15)	2.05 (±0.20)	0.45 (±0.08)	1.89 (±0.04)	0.58 (±0.02)
plants-chemplp	1.78 (±0.17)	0.41 (±0.05)	1.58 (±0.72)	0.36 (±0.34)	1.72 (±0.23)	0.45 (±0.03)	1.54 (±0.12)	0.55 (±0.04)
plants-plp95	2.35 (±0.11)	0.41 (±0.03)	2.14 (±1.08)	0.12 (±0.07)	2.34 (±0.30)	0.38 (±0.05)	2.01 (±0.26)	0.52 (±0.05)
plants-plp	2.18 (±0.12)	0.39 (±0.03)	1.90 (±0.93)	0.15 (±0.08)	2.06 (±0.21)	0.45 (±0.02)	1.87 (±0.10)	0.50 (±0.03)
rdock-solv	4.34 (±0.25)	0.36 (±0.05)	4.54 (±0.51)	0.13 (±0.09)	4.16 (±0.52)	0.38 (±0.08)	3.61 (±0.26)	0.55 (±0.02)
rdock-std	4.28 (±0.10)	0.36 (±0.03)	4.42 (±0.65)	0.12 (±0.08)	4.06 (±0.49)	0.40 (±0.08)	3.54 (±0.18)	0.56 (±0.01)
vina-std	2.08 (±0.12)	0.27 (±0.04)	2.40 (±0.27)	0.05 (±0.08)	2.11 (±0.15)	0.28 (±0.11)	1.92 (±0.05)	0.37 (±0.03)
**Average**	2.41 (±0.12)	0.39 (±0.04)	2.36 (±0.66)	0.18 (±0.15)	2.38 (±0.23)	0.40 (±0.07)	2.14 (±0.12)	0.53 (±0.05)

Table 2: Predictive performance for nRMSD, (± 1 std.) per docking protocol, for each of the four splits considered.

Protocol	RMSE	Pearson's R	RMSE	Pearson's R	RMSE	Pearson's R	RMSE	Pearson's R
	random		ligand scaffold		protein classes		protein classes balanced	
autodock-ga	5.06 (±0.43)	0.53 (±0.04)	4.74 (±1.54)	0.45 (±0.3)	5.18 (±0.21)	0.48 (±0.06)	4.75 (±0.2)	0.63 (±0.03)
autodock-lga	6.87 (±0.26)	0.52 (±0.03)	5.62 (±2.92)	0.23 (±0.15)	7.23 (±0.37)	0.45 (±0.04)	6.65 (±0.35)	0.59 (±0.04)
autodock-ls	3.43 (±0.27)	0.15 (±0.13)	2.85 (±0.92)	0.26 (±0.37)	3.32 (±0.35)	0.25 (±0.14)	3.21 (±0.15)	0.29 (±0.06)
glide-sp	4.16 (±0.1)	0.43 (±0.02)	5.02 (±1.39)	0.22 (±0.14)	4.29 (±0.38)	0.41 (±0.07)	3.49 (±0.16)	0.58 (±0.03)
gold-asp	6.85 (±0.11)	0.56 (±0.02)	7.72 (±0.39)	0.3 (±0.17)	7.03 (±0.47)	0.52 (±0.06)	5.95 (±0.09)	0.68 (±0.01)
gold-chemscore	6.78 (±0.1)	0.54 (±0.02)	7.48 (±0.89)	0.28 (±0.15)	7.04 (±0.37)	0.49 (±0.03)	5.86 (±0.13)	0.67 (±0.02)
gold-goldscore	7.45 (±0.19)	0.53 (±0.02)	8.81 (±0.77)	0.39 (±0.31)	7.57 (±0.33)	0.5 (±0.05)	6.39 (±0.2)	0.66 (±0.02)
gold-plp	6.78 (±0.09)	0.57 (±0.01)	7.12 (±0.9)	0.32 (±0.18)	7.02 (±0.51)	0.52 (±0.06)	5.78 (±0.17)	0.7 (±0.02)
plants-chemplp	5.5 (±0.12)	0.48 (±0.01)	5.39 (±1.75)	0.15 (±0.09)	5.44 (±0.31)	0.48 (±0.06)	4.84 (±0.09)	0.63 (±0.02)
plants-plp95	4.91 (±0.09)	0.48 (±0.02)	6.03 (±0.29)	0.09 (±0.1)	5.08 (±0.14)	0.47 (±0.06)	4.39 (±0.09)	0.63 (±0.02)
plants-plp	5.07 (±0.15)	0.47 (±0.02)	6.05 (±0.6)	0.12 (±0.09)	5.11 (±0.22)	0.48 (±0.06)	4.52 (±0.08)	0.63 (±0.01)
rdock-solv	4.94 (±0.17)	0.42 (±0.03)	4.95 (±0.93)	0.17 (±0.09)	4.76 (±0.33)	0.45 (±0.07)	4.36 (±0.23)	0.59 (±0.02)
rdock-std	4.81 (±0.15)	0.43 (±0.03)	4.36 (±1.57)	0.17 (±0.09)	4.71 (±0.38)	0.44 (±0.08)	4.33 (±0.19)	0.59 (±0.03)
vina-std	0.94 (±0.04)	0.1 (±0.04)	0.89 (±0.08)	0.64 (±0.44)	0.94 (±0.07)	0.15 (±0.01)	0.9 (±0.06)	0.31 (±0.35)
**Average**	5.25 (±0.16)	0.44 (±0.03)	5.5 (±1.07)	0.27 (±0.19)	5.34 (±0.32)	0.44 (±0.06)	4.67 (±0.16)	0.58 (±0.05)

Table 3: Ligand-centric evaluation for the $\mathrm{RMSD_{min}}$ (± 1 std.) metric and the four different proposed split types in this study.

Split type	Pearson's R	RMSE
random	0.45 (±0.01)	2.20 (±0.06)
ligand scaffold	0.49 (±0.2)	2.19 (±0.63)
protein classes	0.48 (±0.03)	2.03 (±0.19)
protein classes balanced	0.54 (±0.01)	1.82 (±0.07)

Table 4: Ligand-centric evaluation for the nRMSD(± 1 std.) metric and the four different proposed split types in this study.

Split type	Pearson's R	RMSE
random	0.55 (±0.01)	4.88 (±0.09)
ligand scaffold	0.57 (±0.15)	5.77 (±0.6)
protein classes	0.57 (±0.04)	4.74 (±0.26)
protein classes balanced	0.62 (±0.01)	4.09 (±0.06)

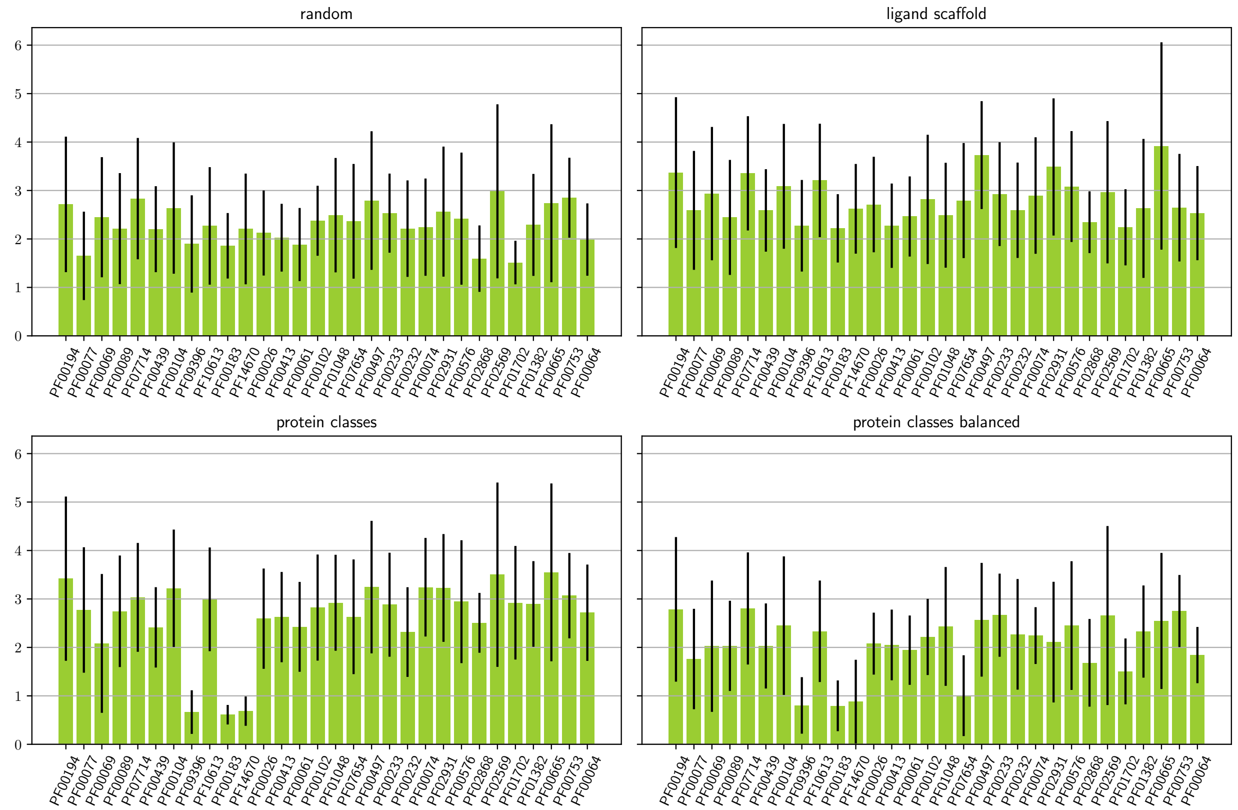

Figure 1: Average RMSE for the RMSD_{ave} metric for all types of splits disaggregated into the 30 most populated PFAM families in the PDBbind refined dataset.
